# Supplementary material for: Adaptive Filtering Framework to Remove Nonspecific and Low-Efficiency Reactions in Multiplex Digital PCR Based on Sigmoidal Trends
Source: Anal Chem. 2022 Oct 3;94(41):14159–68. doi: 10.1021/acs.analchem.2c01883 (PMC9583074; doi:10.1021/acs.analchem.2c01883)
Supplement: Supplementary file 1 — ac2c01883_si_001.pdf [file ac2c01883_si_001.pdf]

Supplementary Information

An adaptive filtering framework to remove non-specific and low-efficiency reactions in multiplex digital PCR based on sigmoidal trends

Luca Miglietta[1,2,‡], Ke Xu[1,2,‡], Priya Chhaya[2], Louis Kreitmann[1], Kerri Hill-Cawthorne[1], Frances Bolt[1], Alison Holmes[1], Pantelis Georgiou[2] and Jesus Rodriguez-Manzano[1,*]

[1] *Department of Infectious Disease, Faculty of Medicine, Imperial College London, U.K., W12 0NN*

[2] *Department of Electrical and Electronic Engineering, Faculty of Engineering, Imperial College London, U.K., SW7 2AZ*

[*]*Corresponding Author: j.rodriguez-manzano@imperial.ac.uk*

Supplementary Information

Table S1 S-2
Figure S1 S-2
Figure S2 S-3
Section S1 S-4

	Precision (%)		Sensitivity (%)		F1-score	
	KNN	RF	KNN	RF	KNN	RF
bla_{NDM}	65.5	80.0	83.7	94.4	0.73	0.87
bla_{IMP}	67.8	75.3	96.0	97.8	0.80	0.85
$bla_{\mathrm{OXA\text{-}48}}$	84.4	94.8	52.8	70.6	0.65	0.81
Accuracy (%)					71.7	83.9

Table S1: Curve-level performance comparison between the original ACA method (45 cycles + KNN) and the proposed 5 parameters + Random Forest method, before applying AMF.

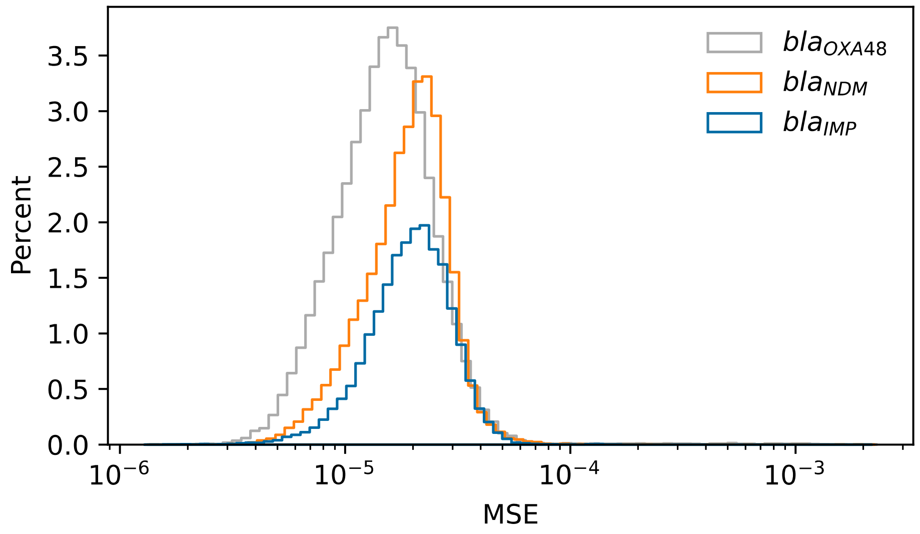

Figure S1. Mean Squared Error distribution. Mean Squared Error (MSE) is used to monitor the fitting performance and the quality/correctness of fitted parameters. As shown in the figure, for the majority of curves a MSE smaller than 10-4 can be observed, indicating good fitting considering the fluorescence values ranged between 0 and 1.

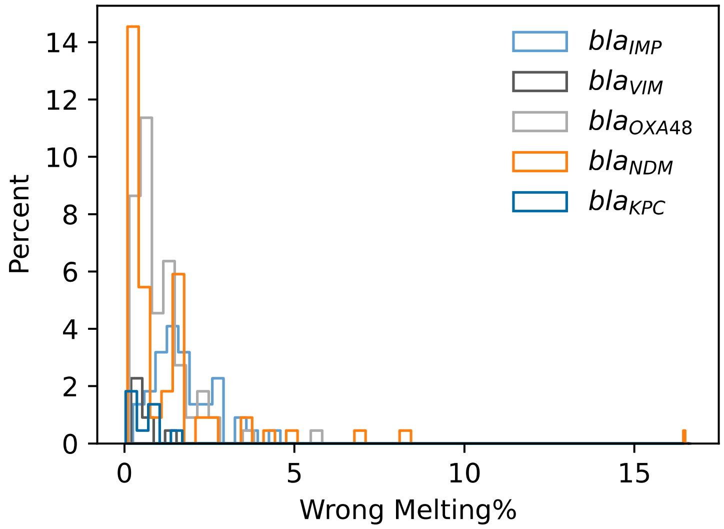

Figure S2. Outlier distribution in clinical samples. On the x-axis the percentage of the wrong melting calculated from our algorithm and on the y-axis the outlier percentage.

Section S1

Assay Design Strategy. Design and in-silico analysis were conducted using GENEious Prime 2020.1.2 (https://www.geneious.com). Primer features were analysed through IDT OligoAnalyzer software (https://eu.idtdna.com/pages/tools/oligoanalyzer) using the J. SantaLucia thermodynamic table for melting temperature (T_m) evaluation, hairpin, self-dimer, and cross-primer formation (https://www.thermofisher.com/uk/en/home/brands/thermo-scientific/molecular-biology/molecular-biology-learning-center/molecular-biology-resource-library/thermo-scientific-web-tools/multiple-primer-analyzer.html). The Tm of the amplification product of each gene was determined by Melting Curve Predictions Software (uMELT) package (https://dna-utah.org/umelt/umelt.html).
